# Adverse drug reactions to antiretroviral therapy during the early art period at a tertiary hospital in Ghana

**DOI:** 10.11604/pamj.2014.18.25.3886

**Published:** 2014-05-07

**Authors:** Margaret Lartey, Abena Asante-Quashie, Ama Essel, Ernest Kenu, Vincent Ganu, Alfred Neequaye

**Affiliations:** 1 Department of Medicine and Therapeutics, University of Ghana Medical School, Accra, Ghana; 2 Fevers Unit, Korle Bu Teaching Hospital, Korle Bu, Accra, Ghana; 3 International Organization of Migration, Accra, Ghana

**Keywords:** Adverse Drug reaction, HAART, HIV, Female sex, Anaemia, CD4 cells

## Abstract

**Introduction:**

Antiretroviral therapy (ART) has reduced HIV morbidity and mortality worldwide but has many adverse effects. These adverse drug reactions (ADRs) lead to discontinuations, disease progression or treatment failure. We explored the types and risk factors for ADRs in a cohort starting ART in a teaching hospital in Accra, Ghana where the main regimens used were a combination of nucleotide and non nucleotide reverse transcriptase inhibitors.

**Methods:**

A Cross-sectional retrospective study was conducted reviewing data of 2042 patients initiated on HAART from 2003 to 2007. Univariate analysis was done for the dependent and independent variables. Stepwise logistic regression procedures were used to model the effect of gender on the development of ADRs controlling for other variables like age, marital status, weight at baseline and CD4 at baseline.

**Results:**

The period prevalence of ADRs was 9.4%. The two most common adverse reactions were anaemia and diarrhoea. Female sex was a statistically significant independent predictor of an adverse drug reaction (AOR: 1.66, p = 0.01, CI: 1.16-2.36). CD4 counts 250 cells/mm3 or more was significantly associated with the occurrence of an ADR. The occurrence of anaemia in females was statistically significant compared to males.

**Conclusion:**

Adverse drug reactions were less common than expected, anaemia was the commonest ADR. Female sex and high CD4 counts >250mm3 were predictors of ADRs whereas females were significantly more likely to develop anaemia than males. Recommendations were made for interventions to prevent and also mitigate the high levels of anaemia especially among women in the ART scale up.

## Introduction

The advent of highly active antiretroviral therapy (HAART) has resulted in significant decreases in HIV-related morbidity and mortality in both the developed and developing world [1–3] and HAART has been touted as one of the greatest breakthroughs in the response to the HIV pandemic. HAART may be modified or interrupted as a result of many reasons, key among which are adverse effects and virological failure [4–7]. The adverse effects may in themselves result in virological failure or disease progression as a result of sub optimal dosing or treatment interruption. In a study by Monforte et al [4], 21% of those who discontinued therapy did so because of toxicity while Tayal et al found that 28.9% of patients were non compliant due to adverse events [8].

In a study done in India, 90.6% of all the patients on HAART developed an adverse drug reaction and there were 618 episodes in various systems, the abdominal and central nervous systems were the most affected [8]. Luma and colleagues, studying patients in Cameroun found an adverse drug reaction (ADR) prevalence of 19.5% of which 21.2% were due to peripheral neuropathy. Overall 56.1% of ADR were attributed to the use of stavudine (d4T) [9]. Anaemia was observed as an ADR in cohorts on ART, whether or not they took zidovudine (ZDV) [10].

In an effort to scale up HAART to those who needed it most, the WHO in 2003 launched the “3 by 5” initiative with an objective of placing 3 million persons living with HIV on HAART by 2005 [11]. In line with this initiative the World Health Organisation (WHO) developed guidelines on antiretroviral therapy for resource poor countries. The guidelines recommended a combination of two nucleoside reverse transcriptase inhibitors (NRTIs) and one non-nucleoside reverse transcriptase inhibitor (NNRTI) as first-line regimens in resource-constrained settings [12]. Ghana adopted the guidelines and started providing HAART in the public sector in 2003 at subsidized rates of $5 a month.

First line first option drugs for Ghana were Zidovudine (AZT) + Lamivudine(3TC) and Nevirapine (NVP) for women of child bearing age or Efavirenz (EFV) for men and post menoupausal women. Stavudine (d4T) was kept as alternative to Zidovudine.

By June 2007, the HIV Treatment Centre (Fevers Unit) of the hospital had almost 6500 patients on register and 2,042 patients on HAART. An electronic health management information system had been started alongside the antiretroviral treatment programme at the Hospital. Data on all aspects of care were entered into the electronic database by trained data assistants and included demographic information, baseline line and follow up clinic assessments, laboratory results, drug regimens, adverse effects and adherence records.

Determining the types and rates of adverse events is critical to the success of any HAART programme. With limited local data on adverse reactions and having provided HAART for four years it was considered prudent assist the national scale up by studying the pattern of adverse drug reactions in this cohort of patients and to provide recommendations to the National AIDS Control Programme in the early or peri HAART era.

We thus examined the spectrum of clinically significant adverse drug reactions and predictors of adverse drug reactions after the initiation of HAART in HIV-infected Ghanaian individuals.

## Methods

A Cross-sectional retrospective study was conducted reviewing patient data stored in the electronic database of the Fevers Unit of the Hospital. The electronic health management information system had been started alongside the antiretroviral treatment programme at the Hospital. Data on all aspects of care were entered into the electronic database by trained data assistants and included demographic data, baseline line and follow up clinic assessments, laboratory results, drug regimens, adverse effects and adherence records. The study population included all patients attending the Fevers Unit between December 2003 and June 2007 and who were naïve to antiretroviral therapy before the initiation of HAART and with valid data in database. There were 6500 patients on register of which 2042 had been initiated on HAART within the time period under study. Ethical approval was provided by the ethical review committee of the University of Ghana Medical School.

### Statistical Analysis

Descriptive statistics showing means and standard deviations for normally-distributed variables and median and inter-quartile range for variables affected by extreme values were generated. Univariate analysis was done for the dependent and independent variables and chi square test of proportions used to determine any form of association. Stepwise logistic regression procedures were used to model the effect of gender on the development of adverse drug reactions, controlling for age group, marital status, weight at baseline, CD4 count at baseline, educational status at enrolment, and WHO clinical stage. In running the stepwise logistic regression, all the variables were re coded into binary categories. Forward and backward selection procedures were used to include only variables that were significant at the level of 0.05. Goodness of fit of the model was assessed by Pearson's method. All statistical analyses were conducted using STATA Release 8.0.

**Ethics Statement:** this study was approved by the Ethical and Protocol Review Committee of the University of Ghana Medical School. All patient data was anonymized and de-identified prior to analysis.

## Results

### Demographic characteristics

We examined 2,042 HIV-infected patients who had received a minimum of 3 months of first-line HAART records between December 2003 and June 2007 at the KBTH Fevers Unit (Table 1). The mean age was 39.5 yrs (SD: 9.8 yrs) and there was a preponderance of females making 59.5% (1215) of the total population. Majority of the patients, 84% (1719) were within the reproductive age group, 50% (1019) were married and only 30% (580) had post basic education. A total of 2,042 Persons living with HIV (PLHIV) were on HAART; 191 (9.4%) had ever had an adverse drug reaction, while 1,851 (90.7%) had never had an adverse drug reaction.

**Table 1:** Demographic Characteristics of Study Participants

Characteristic (N)	Frequency (%)/mean
Mean Age, years (± SD)	39.5 (9.8)
**Age category (2,042)**	
<15yrs	12 (0.6)
15-49yrs	1,713 (83.9)
>49yrs	317 (15.5)
**Sex (2,042)**	
Male	827 (40.5)
Female	1,215 (59.5)
**Marital Status (2,019)**	
Single	459 (22.7)
Married	1,019 (50.5)
Divorced	233 (11.5)
Widowed	284 (14.1)
Co-habiting	24 (1.2)
**Educational Status (1,955)**	
Nil	216 (11.1)
Primary	214 (11)
JSS*	160 (8.2)
MSLC**	785 (40.2)
Secondary/Technical	417 (21.3)
Tertiary	163 (8.3)
**Religion (1,996)**	
Christian	1,777 (89)
Muslim	197 (9.87)
Traditionalist	7 (0.35)
None	13 (0.65)
Other	2 (0.1)

* JSS-Junior Secondary School

** MSLC-Middle School Leaving Certificate

### Clinical Characteristics

Median CD4 count at baseline was 109 cells/mm3 (IQR: 20 - 182 cells/mm3), being slightly higher for females (122 cells/mm3) than for males (88 cells/mm3). Median weight at baseline was 61 kg (IQR: 53.1 - 69.6). Patients in WHO clinical stage I were 102(5%), stage II 633(31%), WHO clinical stage III made up close to half of all attendants, 1001 (49%) and stage IV 306(15%).

### Adverse drug reactions

The most common adverse reaction in our patients was anaemia, comprising almost half of the total number of episodes (45.6%), with diarrhoea taking a close second place (Table 2). As with all the other adverse drug reactions studied in our data, anaemia was more frequent in females. The difference in occurrence of adverse reactions by gender was statistically significant only for anaemia.

**Table 2:** Frequency of Most Common Adverse Drug Reactions

Adverse Drug Reaction	Number of Episodes (%)
Anaemia	69 (48.6)
Rashes	5 (3.5)
Diarrhoea	54 (38.0)
Pancreatitis	5 (3.5)
Pain/numbness/tingling in extremities	4 (2.8)
Hepatotoxicity	3 (2.1)
Blood in Urine	2 (1.4)
**Total**	**142 (100)**

### Predictors of adverse drug reactions

Female gender was a statistically significant independent predictor of an adverse drug reaction in our data (unadjusted OR: 1.52, p = 0.01; adjusted OR: 1.66, p = 0.01, CI:1.16-2,36). Having a relatively high CD4 count 250 cells/mm3 or more was significantly associated with the occurrence of an adverse drug reaction. Conversely, having a low CD4 count of less than 250 mm3 was actually protective - the odds of an adverse reaction in a patient with a CD4 count of less than 250 cells/ mm3 was about 40% less compared to a patient with a CD4 count of 250 cells/ mm3 or more, both before (p = 0.02) and after (p = 0.01, CI: 0.37-0.88) adjustment for demographic and other clinical variables. A baseline weight of less than 60 kg was also protective compared with 60 kg or more (OR: 0.81; p = 0.19), but this was not statistically significant in the unadjusted analysis, and stopped just short of statistical significance in the adjusted analysis (OR: 0.72; p = 0.05). Extremes of age (i.e., ages falling outside the reproductive age group), level of education, marital status, and WHO clinical stage were not significantly associated with the development of an adverse drug reaction in these patients (Table 3).

**Table 3:** Multivariate analysis of factors predicting the occurrence of adverse drug reactions in HIV and AIDS Patients on HAART

Predictor	Adjusted OR (95% Confidence Interval)	P value
**Gender**		
Male	1.00	
Female	1.66 ( 1.16-2.36)	0.01*
**Age category (yrs)**		
15-49	1.00	
<15, >49	0.98 (0.63-1.51)	0.92
**Level of Education**		
Primary	1.00	
Post primary	1.1 (0.75-1.62)	0.60
**Marital Status**		
Not married	1.00	
Married	0.98 (0.71-1.35)	0.91
**CD4 at baseline (cells/mm3)**		
250 or more	1.00	
Less than 250	0.57 (0.37-0.88)	0.01*
**Weight at baseline (kg)**		
60kg or more	1.00	
Less than 60kg	0.72 (0.52-1.00)	0.05
**WHO clinical Stage**		
Stage 1 or 2	1.00	
Stage 3 or 4	1.23 (0.88-1.73)	0.23

## Discussion

The impact of HAART on the natural history of HIV infection is undeniably positive in terms of overall improved health and clinical outcomes. In spite of this, the occurrence of adverse reactions to HAART may negatively impact quality of life and adherence to treatment, limiting its efficacy [13]. Several studies from the developing world have shown that the occurrence of adverse events was the primary reason for modifying therapy [7, 14].

The spectrum of adverse events is wide and varied, and the exact cause may be difficult to identify. However, class-wide and individual side effects have been described. Nucleoside reverse transcriptase inhibitors can cause mitochondrial toxicity and anaemia; non-nucleoside transcriptase inhibitors are associated with rash and central nervous system disturbance; protease inhibitors cause gastrointestinal effects and metabolic problems including hyperlipidaemia, lipodystrophy, and insulin resistance. The prevalence of particular side effects in resource-poor settings is therefore often linked with the drugs comprising the first-line regimen in that setting as was shown in this study.

Most adverse effects can be determined by appropriate clinical examination for specific symptoms and signs, including fatigue with conjunctival pallor (due to Zidovudine-related anaemia); neuropsychiatric problems (due to Efavirenz toxicity); peripheral wasting (due to Stavudine-related lipodystrophy); [14] and rash (due to Nevirapine, Efavirenz or Abacavir). Anaemia was by far the most common adverse event in our patients. In contrast to the unexpected finding of anaemia in less than a tenth of patients in an Indian study (5.4%) [15], almost half of our patients had clinically significant anaemia. This is not surprising, as HIV-infected patients in Ghana often present with coexisting nutritional deficiencies, concomitant opportunistic infections and other chronic diseases that may precipitate low haemoglobin levels. This high level of background anaemia is in turn exacerbated by the use of Zidovudine, which features prominently in our first-line regimens. Curkendall compared a ZDV cohort to a non ZDV cohort and observed a 3 fold increase in the incidence of anaemia in the ZDV group after 6 months of follow up and a little over 2 fold over 24 months of follow up[10].

Further, female patients in our data were significantly more likely than males to experience anaemia. This may accounted for by the generally lower nutritional status of HIV-infected women. Luma et al in Cameroon found that hospital admissions were usually for anemia in their patients [9]. In a cohort predominantly on d4T, Wabe et al still had 17.39% of all adverse effects attributable to anaemia in Ethiopia [16].

Diarrhoea was a common side effect of treatment in our patients. This was difficult to explain since protease inhibitors were rarely used, and the few patients on PIs had been those relocating from the developed countries. It was however thought that the diarrhea could have been due to opportunistic infections like abdominal tuberculosis, parasitic or helminthic infections and not a drug side effect especially since diarrhea was a presenting symptom in many new patients. Lactic acidosis was very rarely diagnosed and the diarrhea was not considered as part of that diagnoses.

We found a surprisingly low rate of hypersensitivity rash in our patients, despite the frequent use of Nevirapine. This may be explained by the routine use of lead-in dosage of Nevirapine of 200 mg once daily, followed by gradual escalation to the full 200 mg twice daily after 2 weeks. Other cohorts had high levels of rash, 17.83% in a cohort in Ethiopia [16] and 15.2% in a cohort in India using mainly nevirapine like Ghana [15].

In addition, our study found a very low rate of hepatotoxicity compared with other studies in developing countries such as Thailand [14]. This may be due to the relatively infrequent laboratory monitoring of liver enzymes and the tendency to defer ART if baseline liver enzymes were found to be high (i.e. more than two times the upper normal limit). Although current WHO guidelines for resource-limited settings recommend haemoglobin and liver enzymes investigations only if symptoms develop, earlier monitoring may be advisable, especially against the background of high prevalence of Hepatitis B(HBV) infection such as exists in Ghana [17].

In those first four and a half years of HAART, peripheral neuropathy was uncommon in our cohort unlike other cohorts [9]. This was likely as a result of zidovudine being the first line first choice NRTI.

The study found that HIV-infected females were at a significantly higher risk of experiencing an ADR than males. This is in line with a study in India which found significant physiological, immunological, and clinical differences between men and women initiating HAART in a resource-limited setting [18]. Some studies in the developed world have also documented gender differences in the distribution of adverse events. The study by Currier found that in using zidovudine, men were three times more likely to reach the study endpoint than females. Some of the end points were progression, adverse event or withdrawal [19]. This finding continues to be explored as more antiretrovirals are developed and approved for use.

Another finding in our study was that relatively higher CD4 cell counts (250 cells/mm3 or more) were associated with a greater chance of an ADR, compared to lower CD4 counts (less than 250 cells/mm3). This was in contrast to the conclusions arrived at in reviewing the adverse effects of HAART in developing countries [14]. The authors concluded that earlier HAART initiation, before the development of a low CD4 cell count and opportunistic infection, may reduce the incidence of adverse effects.

Careful implementation of protocols designed for regular screening of patients, especially during the initial months of therapy, may detect adverse reactions earlier and help prevent serious or life-threatening consequences. In addition, patients and/or supportive family members or adherence monitors can be educated about adverse effects and taught to recognize them for early management to be instituted.

## Conclusion

The period prevalence of ADRs in the patient cohort was 9.4%, much less than expected. The commonest ADRs were anaemia and diarrhoea. Female sex and high CD4 counts > 250mm3 were predictors of ADRs whereas females were significantly more likely to develop anaemia than males. Recommendations were made for interventions to prevent and also mitigate the high levels of anaemia especially among women in the ART scale up.
